# Vaccine Design from the Ensemble of Surface Glycoprotein Epitopes of SARS-CoV-2: An Immunoinformatics Approach

**DOI:** 10.3390/vaccines8030423

**Published:** 2020-07-28

**Authors:** Noor Rahman, Fawad Ali, Zarrin Basharat, Muhammad Shehroz, Muhammad Kazim Khan, Philippe Jeandet, Eugenie Nepovimova, Kamil Kuca, Haroon Khan

**Affiliations:** 1 H.E.J. Research Institute of Chemistry, International Center for Chemical and Biological Sciences, University of Karachi, Karachi 75270, Pakistan; noorbiochemist@gmail.com; 2 Department of Biochemistry, Hazara University, Mansehra 21120, Pakistan; fawwadali792@gmail.com; 3 Jamil-ur-Rahman Center for Genome Research, PCMD, ICCBS, University of Karachi, Karachi 75270, Pakistan; zarrin.iiui@gmail.com; 4 Department of Biotechnology, Virtual University of Pakistan, Lahore 54000, Pakistan; muhammad.shehroz@vu.edu.pk; 5 Centre for Applied Molecular Biology, University of the Punjab, Lahore 53700, Pakistan; kazimbt@gmail.com; 6 Faculty of Sciences, University of Reims Champagne-Ardenne, CEDEX 2, 51687 Reims, France; philippe.jeandet@univ-reims.fr; 7 Department of Chemistry, Faculty of Science, University of Hradec Kralove, 50005 Hradec Kralove, Czech Republic; eugenie.nepovimova@uhk.cz; 8 Department of Pharmacy, Abdul Wali Khan University Mardan, Mardan 23200, Pakistan

**Keywords:** vaccine, multi-epitopes, antigenicity, allergenicity, coronavirus, pneumonia

## Abstract

The present study aimed to work out a peptide-based multi-epitope vaccine against the severe acute respiratory syndrome coronavirus 2 (SARS-CoV-2). We predicted different B-cell and T-cell epitopes by using the Immune Epitopes Database (IEDB). Homology modeling of the construct was done using SWISS-MODEL and then docked with different toll-like-receptors (TLR4, TLR7, and TLR8) using PatchDock, HADDOCK, and FireDock, respectively. From the overlapped epitopes, we designed five vaccine constructs C1–C5. Based on antigenicity, allergenicity, solubility, different physiochemical properties, and molecular docking scores, we selected the vaccine construct 1 (C1) for further processing. Docking of C1 with TLR4, TLR7, and TLR8 showed striking interactions with global binding energy of −43.48, −65.88, and −60.24 Kcal/mol, respectively. The docked complex was further simulated, which revealed that both molecules remain stable with minimum RMSF. Activation of TLRs induces downstream pathways to produce pro-inflammatory cytokines against viruses and immune system simulation shows enhanced antibody production after the booster dose. In conclusion, C1 was the best vaccine candidate among all designed constructs to elicit an immune response SARS-CoV-2 and combat the coronavirus disease (COVID-19).

## 1. Introduction

The 2019 SARS-like coronavirus (SARS-CoV-2), a member of *betacoronavirus*, emerged in late December 2019, causing pneumonia [1]. *Coronaviruses* are enveloped, large, positive-sense RNA viruses belonging to the family of *Coronaviridae,* that can infect mammals, birds, and humans, causing deadly pneumonia [2]. The *Coronaviridae* family consists of two subfamilies (1) *Coronaviridae*, which contain the genera *alpha, beta, gamma*, and *deltacoronavirus* and (2) *Torovirinae*, which comprise a single genera *Torovirus* as well as unknown genera [3].

Since the start of the twenty-first century, two beta coronaviruses have caused deadly pneumonia in humans. In 2002–2003, the severe acute respiratory syndrome coronavirus (SARS-CoV) emerged from being accountable for an outbreak with a death rate of 10% and spread to 5 continents. In 2012, the Middle East respiratory syndrome coronavirus (MERS-CoV) became prominent in Saudi Arabia and caused repeated outbreaks in humans with a 35% death rate [4,5]. In December 2019, a novel SARS-like coronavirus (SARS-CoV-2) emerged, that caused pneumonia with high morbidity and mortality rates responsible for 3,303,296 infections, 235,290 deaths in 185 countries; Dated 1 May 2020 (https://gisanddata.maps.arcgis.com/apps/opsdashboard/index.html#/bda7594740fd40299423467b48e9ecf6). SARS-CoV, MERS-CoV, and 2019-SARS-CoV-2 are zoonotic and their primary hosts are bats and civets, camel being an intermediate host. However, it was suggested that the SARS-CoV-2 was directly transferred from bats to humans from Wuhan seafood in the Hubei province of China. Other reports revealed the occurrence of human-to-human transmission [1,4,5]. There are four other coronaviruses, HCoV-229E and HCoV-NL63 (alpha coronaviruses) and HCoV-HKU1 and HCoV-OC43 (beta coronaviruses), responsible for mild respiratory tract infections that cause complications or fatalities in elderly immuno-compromised individuals and young children [6]. Currently, there are some antiviral treatments like Chloroquine, Remdesivir, Ribavirin, Lopinavir, traditional Chinese medicine etc., under trial to combat SARS-CoV-2. *In silico* drug screening against viral proteins using natural products, FDA approved drugs, and other antivirals have also been attempted [7]. A whooping 657 clinical trials for various drugs against COVID-19 have been registered till 20 April 2020 (https://clinicaltrials.gov/ct2/results?cond=COVID-19). Although some studies report the use of these drugs in some COVID-19 patients, their concordant status against the disease is yet inconclusive and no satisfactory treatment for COVID-19 exists till to date. It has been proposed that sequence similarity and computational approaches can lead to vaccine design, based on derived epitope and antigen information from surface glycoprotein of SARS-CoV-2 [7]. It has also been proposed that the initial antigenic target of vaccine should be the surface protein since it allows entry of coronavirus into the host [8]. Yuan et al. (2020) [9] isolated a neutralizing antibody, bound to surface protein of a COVID-19 patient, which provides evidence that spike protein produces an antibody response and could be an effective target for vaccine design.

**Figure 1:**
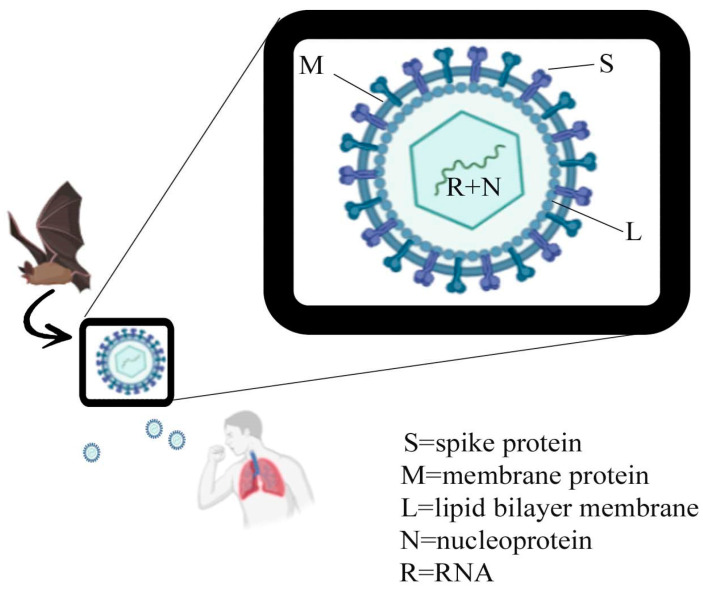
Different proteins and RNA + Nucleoprotein are shown in the structure of the novel SARS-like coronavirus (SARS-CoV-2), originated from the bat and causative agent of COVID-19.

The S viral protein of the SARS-CoV-2 is a single polypeptide chain of 1273 amino acids [1]. The surface (S) glycoprotein of coronavirus showed in Figure 1 contains a homotrimer which is used for virus entry to promote host attachment and fusion of the virus with host cell membranes [7]. Surface glycoprotein is a class I viral fusion protein which represents the leading focal point for vaccine designing as it is the principal antigen of the virus that neutralizes antibodies during infection [2]. It contains two subunits, S1 and S2, generated by the action of host proteases which are bound by intermolecular forces in the pre-fusion conformation. The N-terminal S1 ectodomain is composed of four beta-rich domains, designated as A, B, C, and D with A or B comprises a receptor-bounding domain.

The C-terminal S2 subunit is a transmembrane domain that mediates membrane fusion [10,11]. Currently, no registered vaccine is available for COVID-19, but four vaccines are under trial while many more are under development. Computational epitope mapping is a swift way to add to the knowledge base of the COVID-19 vaccine landscape. Epitopes are the antigenic determinants, located in proteins that have the capability of initiating a cellular immune response, which in turn is arbitrated by T or B cells. T cell epitopes are usually protein antigen-derived peptides presented by major histocompatibility complex (MHC) molecules on antigen-presenting cells. These are recognized by T-cell receptors and hence, called T-cell epitopes. B cell epitopes are capable of binding an antibody. Both of these cells mediate adaptive immunity i.e., develop pathogen-specific memory. This convenes immunological protection. Identification and study of epitopes are therefore of prime interest to scientists working on diagnostic assay development and epitope-based vaccines. *In silico* prediction of epitopes is a swift and economical method that helps sift useful candidates from an ensemble of predicted ones. We adopted a multi-epitope-based strategy for this study due to several benefits of multi epitope-based vaccine design, including rational engineering of the epitopes for better potency, improved safety profiling, and spotlighting of immune responses for conserved epitopes [12]. A chemoinformatics approach for the development of a multi-epitope vaccine, as well as it’s *in silico* cloning and expression against the deadly COVID-19, could provide clues to combat human coronavirus.

## 2. Materials and Methods

### 2.1. Protein Sequence Retrieval

**Figure 2:**
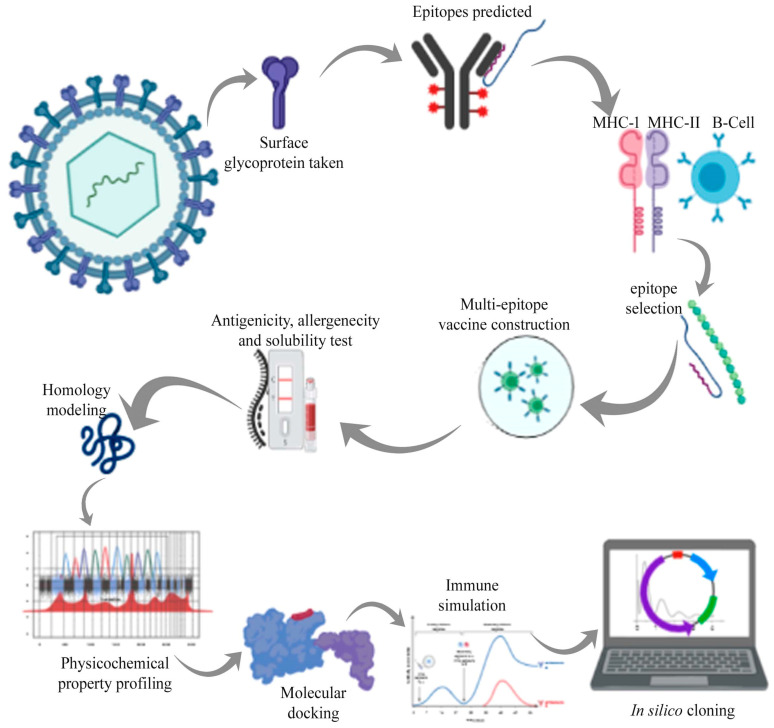
Schematic workflow followed for the multi-epitope vaccine design.

In the current strategy, the art of immunoinformatics approach was used for the development of the multi-epitope-based vaccine. The stepwise analysis followed for the study is depicted in Figure 2. The amino acid sequence (1273 amino acids) of the surface glycoprotein SARS-CoV-2 was retrieved from NCBI (Accession no: AYN64561.1) (https://www.ncbi.nlm.nih.gov/).

### 2.2. Epitope Prediction

For chimeric vaccine development, the surface topology of the glycoprotein was selected to identify immunogenic determinants for the vaccine construct. Identification of immunogenic T-Cells (MHC-I and II) was carried out by using the Immune Epitopes Database (IEDB) (https://www.iedb.org/) [13]. It employs a different prediction method for MHC epitope binding analysis. MHC class I and II molecules were predicted through the Stabilized Matrix Method (SMM) scoring and neutral-network based tool (net MHC-1.1) [14,15,16]. Epitopes were prioritized from the predicted ensemble, based on a threshold value of IC_50_ ≤ 200 nM. B-Cell immunogenic determinants were projected using BCPRED (http://ailab.cs.iastate.edu/bcpreds/), with the default parameters. BCpred predicts linear B-Cell epitopes [17], which are important for stimulating a humoral immune response, which activates B lymphocytes for antibody production [18]. Interferon-inducing epitopes were identified from the MHC-II binding epitopes by using the IFNepitope web server (http://crdd.osdd.net/raghava/ifnepitope/predict.php). IFNepitope predicts regions in the protein sequence or antigen, that cause induction of Interferon-gamma (IFN-gamma) (Dhanda et al., 2013). The overlapped epitopes were submitted to IFNepitope web server and IFN-gamma was predicted based on Support Vector Machine (SVM) and model was predicted by selecting IFN-gamma versus non-IFN-gamma [19].

### 2.3. Epitopes Selection and Vaccine Construction

The B-cell and T-cell epitopes are immunodominant and crucial to enhance neutralizing-antibody response when exposed to toxins and contagions. Identification of B-cell epitopes is indispensable for epitope-based vaccine design and development [20]. MHC encodes cell surface proteins which are vital for the adaptive immune system, including MHC-II binding interferon-inducing epitopes. B-Cell, MHC-I, MHC-II, and IFN epitopes were ranked based on cut off values and compared manually for overlapped peptides. Four overlapped and high score epitopes were selected for vaccine constructs. Universal T-helper epitopes, PADRE (Pan HLA-DR reactive epitope) and five different adjuvants were added. PADRE is projected as a simple carrier epitope, useful in making synthetic or recombinant vaccines and known to have enhanced response when coupled with adjuvant [21]. Adjuvant increases the immune response of a vaccine, leading to more antibody production and reduces the quantity of antigen input. Adjuvants used in this study were mined from literature and included heparin-binding hemagglutinin (HBHA) from *Mycobacterium* sp., Beta defensin, Ribosomal protein, and flagellin. HBHA has been implicated as a vaccine adjuvant, with demonstrated usage in antitumor immunotherapy as well [22]. Beta defensin is an antimicrobial peptide with a role in innate immune responses and can initiate cellular immune response [23]. Ribosomal protein has been identified as a non-traditional adjuvant [24], while flagellin activates diverse cell types, which are part of innate and adaptive immunity, promoting cytokine production [25]. Effectiveness of all these adjuvants has been reported in the literature and they initiate cellular immune responses, leading to antigen-specific immune response stimulation. All of these are TLR agonists and it is reported that binding with TLRs shows enhanced results in activating immune responses because TLRs conduct signaling and activate the innate and adaptive immune response [26]. After addition of PADRE and the adjuvant, the vaccine constructs were admixed in different combinations with the help of GGGS, HEYGAEALERAG, and EAAAK linkers [27,28].

### 2.4. Determination of Antigenicity, Allergenicity, Toxicity, and Solubility

Antigenicity of the constructed vaccine was predicted through Vaxijen v2.0 (http://www.ddg-pharmfac.net/vaxijen/VaxiJen/VaxiJen.html) [29] and predicted antigenic peptides (http://imed.med.ucm.es/Tools/antigenic.pl) was used to determine the antigenicity of the predicted epitopes. An online server, AlgPred (http://crdd.osdd.net/raghava/algpred/) was used to determine the allergenicity of the constructs with −0.4 threshold value [30]. The solubility of the constructed vaccine upon expression in *Escherichia coli* was determined by using SOLpro (http://scratch.proteomics.ics.uci.edu/) [31].

### 2.5. Physiochemical Properties

Expasy Protparam (https://web.expasy.org/protparam/) was used to predict physicochemical properties like amino acid composition, atomic composition, molecular weight, theoretical pI, extinction coefficient, estimated half-life, aliphatic index, grand average of hydrophobicity (GRAVY), and instability index of the vaccine constructs [32].

### 2.6. Homology Modeling and Model Validation

Homology models were built for the vaccine construct, using the SWISS-MODEL (https://swissmodel.expasy.org/) online server, which is the most reliable server for protein modeling [33]. The 3D model structure was further validated using RAMPAGE Ramachandran plot (http://mordred.bioc.cam.ac.uk/~rapper/rampage.php).

### 2.7. Molecular Docking and MD Simulation

Molecular docking is used to predict the possible binding orientation of the vaccine constructs [34,35]. TLR7/8 complex (TLR8 PDB ID:3w3g) was downloaded from Protein Databank (PDB) (https://www.rcsb.org). Online servers PatchDock (https://bioinfo3d.cs.tau.ac.il/PatchDock/), HADDOCK webserver (https://doi.org/10.1007/978-1-4939-0366-5_12), and FireDock (http://bioinfo3d.cs.tau.ac.il/FireDock/) were used for molecular docking and docking refinement, respectively. FireDock refines and re-scoring the docked complexes and ranks the complexes based on binding score and global binding energy [36]. An online server CABS-flex (http://212.87.3.12/CABSflex2/job/7e5d9345a1a8235/) was used for molecular dynamics simulation, to check the stability of the complex.

### 2.8. Immune Simulation of Vaccine Construct

Immune simulation of vaccine construct C1 and positive control (construct having no adjuvant) using web server C-ImmSim (http://kraken.iac.rm.cnr.it/C-IMMSIM/) was done. It is an agent-based model implementation where information about the humoral as well as the cellular response of the mammalian immune system, invoked by antigen at the cellular level is obtained. The simulation time steps 1, 42, and 126 h (8 h correspond to one cell division cycle in real life according to Kaba et al. (2018)) [37] were chosen along with homozygous host haplotypes (HLA-A*0101, HLA-A*0201, HLA-B*0702, HLA-B*3901, HLA-DRB1*0101, and HLA-DRB1*0401), while other general simulation parameters were: random seed: 1234, simulated volume: 10, simulation steps 1000, Adjuvant = 100 [38]. The comparison was drawn between positive control and construct and results were interpreted.

### 2.9. Codon Optimization and In Silico Cloning

A codon optimization approach was used to enhance recombinant protein expression. Codon optimization is important because degeneracy of genetic code permits most of the amino acids to be encoded by multiple codons. Java codon adaptation index (JCAT) (http://www.jcat.de/) and EMBOSS Backtranseq (www.ebi.ac.uk/) were used in the codon system of *E. coli* to obtain the codon adaptation index (CAI) values and GC contents [39].

The optimized sequence of the final vaccine construct was inserted in a vector for expression, by using the Snapgene tool available at (https://www.snapgene.com/free-trial/). A compatible plasmid vector pET-28a (+) was used to integrate the optimized sequence and clone the constructed chimeric vaccine. pET-28a (+) is for expression of N-terminally 6 × His-tagged proteins and usually, N-terminal tags are advantageous over C-terminal tags, leading to enhanced purification, protein recovery, and stronger response.

## 3. Results

The T cell, B cell, and IFN-inducing epitopes of the antigenic surface glycoprotein were predicted with the help of Immune Epitope Database (IEDB) and IFNepitope servers. The predicted T cell epitopes were able to bind with MHC-1 and MHC-2 molecules to activate the adaptive immune response in the host. We searched the overlapped epitopes as shown in Table 1 (the epitopes bind with both MHC molecules, B cells, and IFN-gamma) to take advantage and produce humoral, cell-mediated immune responses and induce interferons which were our ultimate objective.

Five different constructs were built by the integration of two antigenic peptides and several adjuvants and linkers. There were a lot of possible arrangements of segments but since the analysis of such a huge number of constructs was impossible, we designed these five constructs as shown in Table 2, as samples for our study. The sequence of these constructs differed from each other according to the adjuvant used and also the arrangement order of the constituent segments. The linkers “EAAAK” (blue) links the adjuvant (black) in the constructs. The GGGS and HEYGAEALERAG (grey) link the epitopes. Immunogenic epitopes (red) and the immune enhancer adjuvant, PADRE, as well as linker sequences were inserted as shown in Table 2**.**

All the five vaccine constructs were checked for allergenicity, antigenicity, and solubility by Algpred server, Vexijen v2.0 [40] (Designed by Medical University Sofia, Bulgari and The Jenner Institute, Oxford University, Compton, Berkshire, RG20 7NN, UK) and SOLpro, respectively as shown in Table 3. The least allergenic and most antigenic C1 vaccine construct was selected as a promiscuous vaccine to elicit host immune response.

**Figure 3:**
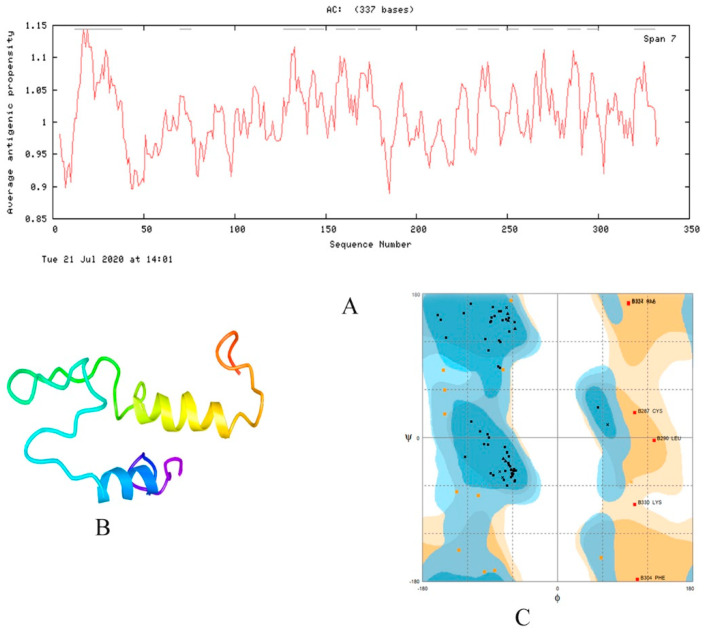
(**A**) The average antigenic propensity (1.0098) of vaccine construct-1 (C-1). Grey lines above the graph show regions with increased antigenicity. (**B**) 3D structure of the final vaccine, which contains two right-handed alpha helices (DodgerBlue and Yellow) and coil and random coils (DeepSkyBlue, LawnGreen, and OrangeRed). (**C**) Ramachandran plot of the modeled vaccine construct. The number of residues in favored regions were 63 (77.8%), 12 were in allowed (14.8%) and 6 (7.4%) were outliers.

Physiochemical properties of all vaccine constructs were predicted by the ProtParam server as shown in Table 4. The molecular weight of the vaccine constructs was predicted between 23 kDa to ~36 kDa. The GRAVY score [41] was found between the values −0.14 to −0.45, which shows a hydrophilic nature of the vaccine constructs. The high aliphatic index score (64.13 to 80.42) indicates their stability at several temperatures. All vaccines constructs showed good instability index (<40) except construct 3 (C3) and these stable constructs can initiate an immunogenic reaction [42]. The estimated half-life of construct-1 (C-1) is 1 h in mammalian reticulocytes (*in vitro*), 30 min in yeast (*in vivo*), and more than 10 h in *Escherichia coli* (*in vivo*). Antigenic propensity was calculated for 337 residues of vaccine construct. It was above one for majority residues and considerable variation was seen at some places, with decreased antigenicity near residue 50, 175, and 310. High span regions with enhanced antigenicity were 13 in total (Figure 3A) and average antigenicity was slightly above 1, i.e., 1.0098.

These tertiary structures are considered as critical regarding their interactions with other proteins and molecules within the cell. Tertiary structures of the constructed vaccines were generated by using the SWISS-MODEL server. PatchDock, HADDOCK web server, and FireDock were used for molecular docking and refinement, respectively, and then validated by RAMPAGE. The final 3D modeled structure of the vaccine construct is shown in Figure 3B and the Ramachandran validation plot is displayed in Figure 3C.

**Figure 4:**
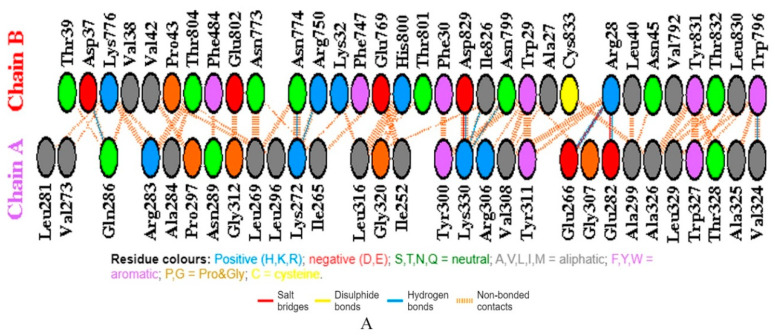

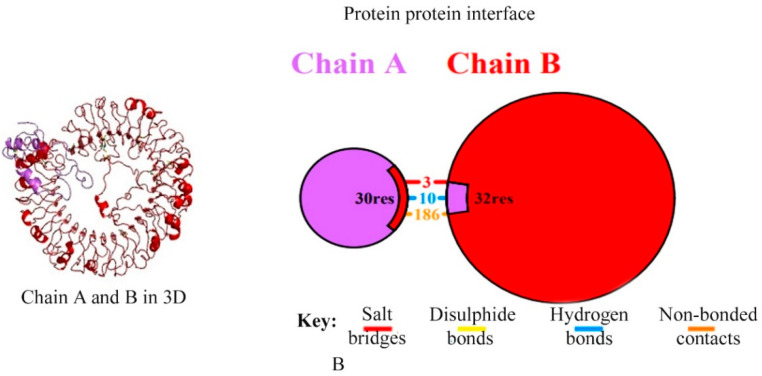
(**A**) The number of H-bond lines between any two residues indicates the number of potential hydrogen bonds between them. For nonbonded contacts, which can be plentiful, the width of the striped line is proportional to the number of atomic contacts. (**B**) Schematic diagram of interactions between protein chains. Interacting chains are joined by colored lines, each representing a different type of interaction, as per the key above. The area of each circle is proportional to the surface area of the corresponding protein chain. The extent of the interface region on each chain is represented by the black wedge whose size signifies the interface surface area. Chain A refers to a vaccine construct while chain B is toll-like receptor.

A vaccine able to bind to different HLA allelic proportion of the human population is most important for its proper function inside the host. The docking of all vaccine constructs was performed with TLR7, TLR8, and TLR 4/MD2 complexes (PDB ID 2Z65). After analyzing all vaccine constructs, we finalized the vaccine construct (C-1) based on different physiochemical properties and docking scores. The C-1 TLR7 complex has good global binding energy score (−65.88 Kcal/mol) obtained from PatchDock as shown in Table 5. Therefore, we considered construct 1, as a promising vaccine against the 2019 novel Coronavirus (2019-nCoV). Further molecular docking of the vaccine was performed with the modeled TLR7 using the HADDOCK web server. The docking results revealed that the vaccine showed a strong binding affinity with the TLR receptor indicated in Table 6. Molecular interaction formed between vaccine and TLR can be seen in Figure 4, which shows 3 salt bridges, 10 hydrogen bonds, and 156 non bonded interactions between both molecules. Root mean square fluctuation (RMSF) of chain A was observed to be maximum at position 256. This residue was not involved in direct interaction with another chain. However, the nearby residue at position 252 was involved in non-bonded contact formation with Glu at position 769, which means that the interaction had an impact on RMSF. Similar observations were made for chain B where most fluctuations were observed at residue 122, 271, 354, 488, and near 818, but these residues were not directly involved in bond formation with chain A. Nearby residue interaction distorts geometry and causes fluctuation of main-chain atom coordinates from alpha carbon backbone.

**Figure 5:**
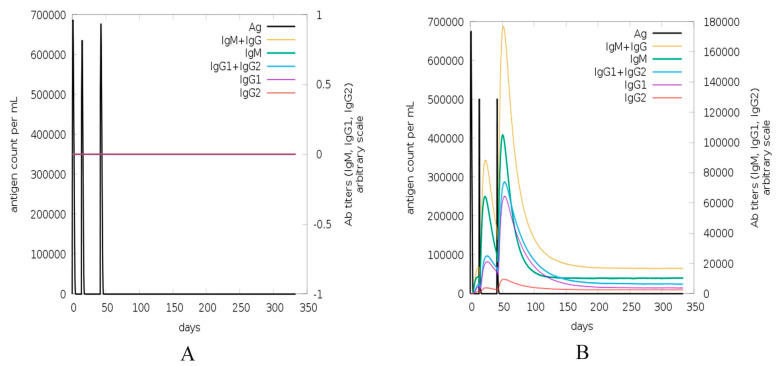

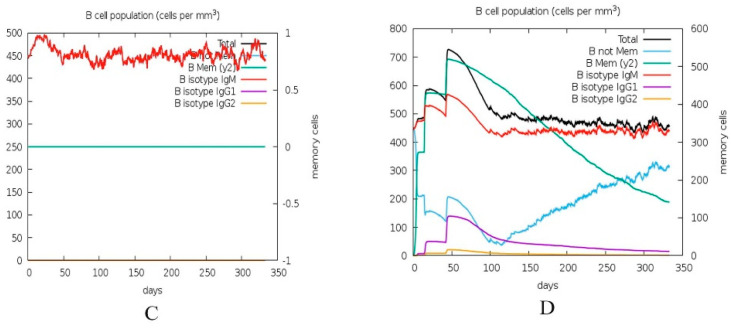
(**A**) Antigen and immunoglobulins of control, (**B**) antigen and immunoglobulins of construct C1 with antibodies sub-divided per isotype. (**C**). B lymphocytes showing total count, memory cells (isotypes IgM, IgG1, and IgG2) for control. (**D**). B lymphocytes showing total count, memory cells (isotypes IgM, IgG1, and IgG2) for construct C1.

**Figure 6:**
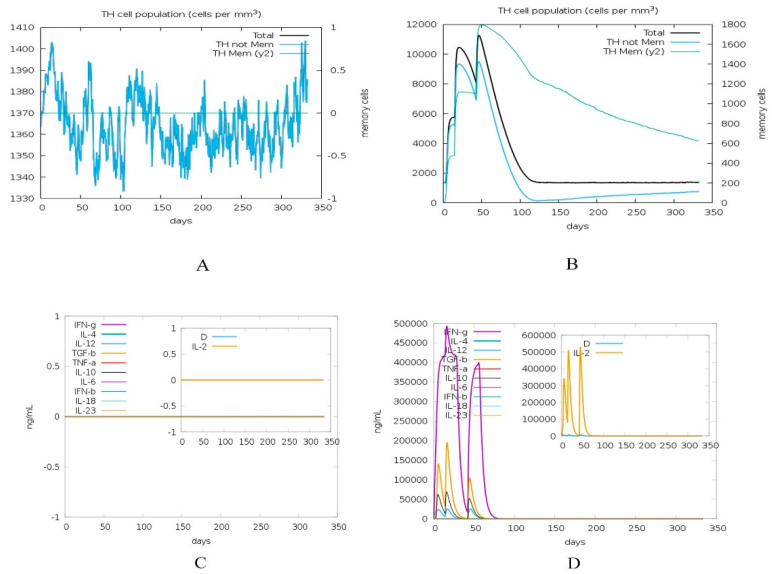
(**A**) The plot shows total and memory counts of CD4 T-helper lymphocytes for control. (**B**) The plot shows total and memory counts of CD4 T-helper lymphocytes for construct C1. (**C**). The concentration of cytokines and interleukins for control with D in the inset plot representing danger signal. (**D**). The concentration of cytokines and interleukins for construct C1.

The immune simulation was carried out using C-ImmSim, which accounts for both B-cell and HLA class I/II epitope prediction as well as the interaction of the T-cell receptor with the peptide-HLA complexes. This prediction is made through an agent-based representation of immune cells and the interaction potential of amino acids [43]. Booster doses were given because initial immunization response is comparatively sluggish with low antibody concentration, while the booster immunization is pretty fast, with high-affinity antibody, mostly IgG production in bulk. This is evident for our vaccine construct simulation (Figure 5B), where IgG increased after the booster dose. The positive control showed no change in antibody titer in the absence of the adjuvant. Total count of B lymphocytes and non-memory cells, as well as isotypes IgG1 and IgG2, were varied for C1 vs. control. CD4 T-helper lymphocytes count, especially memory cell count shown in green, is very high in C1 (Figure 6B) compared to control. The concentration of cytokines and interleukins is also considerably increased after C1 administration (Figure 6D) compared to the control.

**Figure 7:**
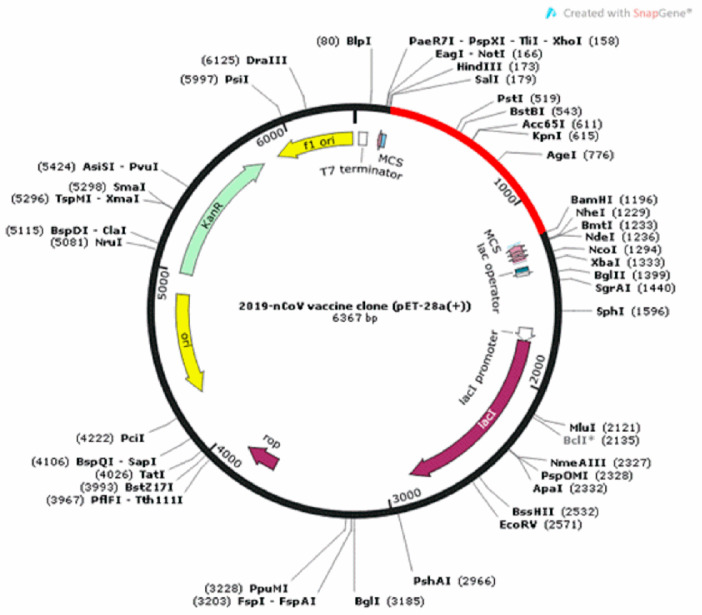
Expression of the final vaccine gene (Red) in a restriction cloning vector pET28a in *E. coli* host.

To analyze the expression and to clone the constructed vaccine inside a suitable vector, we translate back the protein amino acids sequence to a cDNA nucleotide sequence using the Java codon adaptation tool and EMBOSS Backtranseq. The final vaccine construct showed 54.0% GC content when analyzed by codon optimization tool. The GC content was in the normal range (30–70%). The codon optimization index (CAI) value was predicted 1.0, which indicates high expression in *E. coli* indicated in Figure 7.

## 4. Discussion

**Figure 8:**
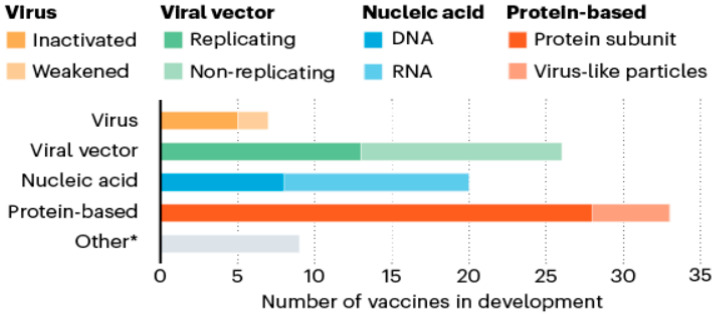
An array of vaccines: eight different approaches including viral-inactivated & weakened, viral vector-based replicating & non-replicating, protein-based subunit & virus-like particles are used for the development of SARS-CoV-2 (source: Nature Briefing).

Severe acute respiratory syndrome coronavirus 2 (SARS-CoV-2) is a new and challenging virus that causes severe infection in humans. The complete genome sequence of the pathogen was reported by [1] on 29 January 2020. In the present study, we took advantage of the available genomes of SARS-CoV-2 to predict an immunogenic, multi-epitope vaccine with different immune enhancer adjuvants and linker sequences. Different approaches, like an inactivated or weakened virus, replicating or non–replicating viral vector, DNA or RNA and protein-based are used for SARS-CoV-2 vaccine development, as indicated in Figure 8 [44].

**Figure 9:**
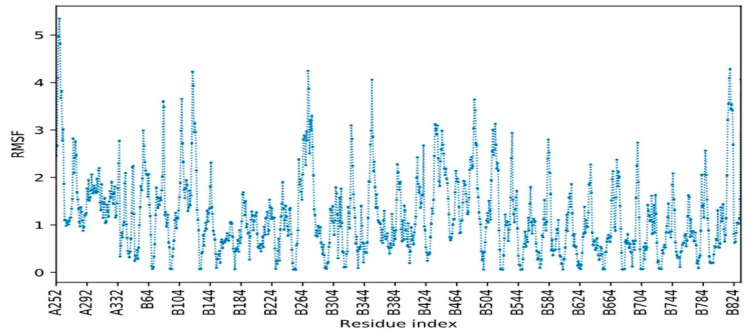
RMSF of chain A (vaccine construct) and B (TLR receptor) of the docked complex. Residue starting with A belong to chain A, while those starting with B reflect chain B residues.

Twenty-eight teams are working on vaccines with viral protein subunits, most of these groups are focusing on spike protein. We also selected spike protein to develop potent vaccine construct from the different immunogenic determinant of the spike glycoprotein of SARS-CoV-2. The immunogenic, multi-epitope, subunit vaccine was generated from MHC-I, MHC-II alleles, B-Cell and IFN-inducing epitopes of the surface glycoprotein (S) [16]. Only peptides having IC50 values < 200 were considered as effective peptides. All the selected epitopes were merged with different adjuvants, linkers, and Pan-DR sequence epitopes (PADRE). PADRE sequence is responsible for the reduction in polymorphism in HLA DR molecules in the population [45]. We have also used the G-rich linker GGGS which enhances the immunogenicity of the vaccine inside the host [46]. Five vaccine constructs (C1, C2, C3, C4, C5) were made. All the vaccine constructs were further analyzed for antigenicity, solubility, and allergenicity. We also predicted the physicochemical properties of vaccines and shortlisted the one vaccine construct, C1, which showed good properties among others with 1.0093 average antigenic propensity as shown in Figure 3**.** As the molecular weight of the candidate vaccine C1 is 35.9 kDa, this makes it possible to predict its solubility during expression, such a molecular weight being able to trigger an immune response. The theoretical pI [47] is predicted to be 6.0, indicating that the protein is acidic. In addition, the predicted instability index [48] indicates that the vaccine peptide will be stable upon expression, thus further firming its potential for use. The aliphatic index indicates good hydrophobicity [41,47]. All the above properties support our constructed vaccine as promiscuous against the SARS-CoV-2. The 3D model of the construct was built with the online server and validated by Ramachandran plot analysis. Furthermore, the docking analysis of the final vaccine C1 was done with different toll-like receptors (TLR4, TLR7, and TLR8). TLR4 has been involved in the recognition of viral structural and non-structural proteins leading to inflammatory cytokine production [49]. TLR4-activating viral proteins include the RSV fusion protein (F), the EBOV glycoprotein, the vesicular stomatitis virus glycoprotein (VSV G), and the dengue virus (DENV) [50]. TLR7 and 8 are key players in antiviral responses. TLR7-specific agonists activate plasmacytoid dendritic cells (pDCs) and B cells and mainly induce IFN-α and IFN-regulated cytokines. TLR8-specific agonists activate myeloid DCs, monocytes, and monocyte-derived DC, leading primarily to the production of proinflammatory cytokines and chemokines, such as TNF-α, IL-12, and MIP-1α [51]. The ability of TLR7 and TLR8 agonists to activate DCs and thus elicit Th1 and CD8+ T cell responses can be exploited to enhance the efficacy of vaccination [52,53]. Activation of TLR7/8 triggers different signaling pathways in human monocytes that halt viral pathogenesis by the induction of interferons (TFNs). In the innate immune system, plasmacytoid dendritic cells TLR7 and TLR9 trigger induction of proinflammatory cytokines and IFN-α/β. The docking results showed good global energy scores for TLR7 that we used during the analysis for the vaccine C1, which was the indication of eliciting an optimal immune response against the SARS-CoV-2. The docked complex when simulated using CABS-flex (2) dynamics revealed that both molecules remained stable with minimum fluctuations RMSF. The flexibility of both molecules can be seen in the trajectory in Figure 9.

Simulation by C-ImmSim through a virtual injection was done for HLA allele-specific (HLA-A*0101, HLA-A*0201, HLA-B*0702, HLA-B*3901, HLA-DRB1*0101, and HLA-DRB1*0401) outcome of the vaccine construct. This was to check the proficiency of construct to the adaptive immune system (reliant on T-cell receptor diversity, through V, J arrangements of the alpha and V, D, J arrangements of the beta unit in the thymus). The output of the simulator was a graphical illustration of the total count of lymphocytes, division amid isotypes, antibody, and cytokine concentration. Cells may bind or move and follow environmental harmonized behavior [43,54]. The decrease in antigen count was observed for C1 after 50 days and diversity finally reached zero around the 70th day of injection (Figure 6D). The antibodies titers (IgM and IgG1 + IgG2) showed a high peak after booster doses of C1 injection. Greater IgM production is required for enhanced primary immune response, resulting in augmentation of B cell population and additional antibodies responsible for secondary and tertiary immune reactions [12]. Overall B cell population was found highest around the 50th day (in cells per mm^3^) of injecting C1, before plateauing off. Increased CD4 T-helper cell population has a vital role in evoking protection and was evoked after injection (Figure 6B). Nevertheless, this prediction is preliminary and the study of the construct with diverse HLA-alleles is suggested as antigen-specific immune response relies not only on age, dose, the time interval of booster dose, but mostly immunogenetics of population.

Vaccine construct C1 was cloned *in silico* with the help of Snapgene in the most suitable plasmid vector pET28a (+) by restriction enzymes SalI and BamHI to check its expression and purification in the bacterial cellular environment. Analysis of the virtual cloning, after codon optimization, validated the stance that translated chimeric vaccine construct appears proficient with enhanced gene expression and is capable of vaccine production at an economical cost.

## 5. Conclusions

In conclusion, we utilized the immunogenic B-cell, T-cell, and IFN-inducing epitopes to generate a peptide-based multi-epitope vaccine from the surface glycoprotein which elicits humoral and cell-mediated immunity, respectively, to eradicate viral particles. Predicted epitopes were merged using appropriate linkers and adjuvants to enhance the immunogenicity of the vaccine. Antigenicity, allergenicity, and solubility, as well as physiochemical properties and tertiary structure analysis, were confirmed. Docking and MD simulation analysis of TLR7 and vaccine were performed, allowing evaluation of the binding affinity and stability of the complex. The immune simulation showed an enhanced antibody titer production and CD4 T-cell count after C1 virtual administration. It also shows that adjuvant has an important role in healthy immune response elicitation. The final vaccine construct was back-translated and *in silico* cloned in a plasmid, which showed effective expression.

## Figures and Tables

**Table 1:** Overlapped (Red) epitope of B-cell, MHC-I, and MHC-II with IC_50_ values.

S. No	Position	Final B-cell Epitope	MHC-I	IC_50_	MHC-II	IC_50_
1	404–424	GDEVRQIAPGQTGKIADYNYK	VRQIAPGQT	31.27	VRQIAPGQT	121
2	673–691	SYQTQTNSPRRARSVASQS	QTQTNSPRR	34.53	QTQTNSPRR	56
3	805–826	ILPDPSKPSKRSFIEDLLFNKV	ILPDPSKPS	23.04	ILPDPSKPS	157
4	14–36	QCVNLTTRTQLPPAYTNSFTRGV	TQLPPAYTN	11.95	TQLPPAYTN	121

**Table 2:** Different vaccine constructs (C1-5). An adjuvant is shown in Black, linker ’EAAAK’ joining adjuvant shown in Blue, linkers ‘GGGS’ ‘HEYGAEALERAG’ joining epitope shown in Grey, epitopes shown in Red, and PADRE sequence shown in Green.

C1 adjuvant = HBHA adjuvant
EAAAKMAENPNIDDLPAPLLAALGAADLALATVNDLIANLRERAEETRAETRTRVEERRARLTKFQEDLPEQFIELRDKFTTEELRKAAEGYLEAATNRYNELVERGEAALQRLRSQTAFEDASARAEGYVDQAVELTQEALGTVASQTRAVGERAAKLVGIELEAAAKAKFVAAWTLKAAAGGGSGDEVRQIAPGQTGKIADYNYKGGGSSYQTQTNSPRRARSVASQSGGGSAKFVAAWTLKAAAGGGSILPDPSKPSKRSFIEDLLFNKVHEYGAEALERAGQCVNLTTRTQLPPAYTNSFTRGVHEYGAEALERAGAKFVAAWTLKAAAGGGS
C2 adjuvant = Beta defensin adjuvant
EAAAKGIINTLQKYYCRVRGGRCAVLSCLPKEEQIGKCSTRGRKCCRRKKEAAAKAKFVAAWTLKAAAGGGSGDEVRQIAPGQTGKIADYNYKGGGSILPDPSKPSKRSFIEDLLFNKVGGGSAKFVAAWTLKAAAGGGSSYQTQTNSPRRARSVASQSHEYGAEALERAGQCVNLTTRTQLPPAYTNSFTRGVHEYGAEALERAGAKFVAAWTLKAAAGGGS
C3 adjuvant = HBHA conserved
EAAAKMAENSNIDDIKAPLLAALGAADLALATVNELITNLRERAEETRRSRVEESRARLTKLQEDLPEQLTELREKFTAEELRKAAEGYLEAATSELVERGEAALERLRSQQSFEEVSARAEGYVDQAVELTQEALGTVASQVEGRAAKLVGIELEAAAKAKFVAAWTLKAAAGGGSSYQTQTNSPRRARSVASQSGGGSQCVNLTTRTQLPPAYTNSFTRGVGGGSAKFVAAWTLKAAAGGGSGDEVRQIAPGQTGKIADYNYKHEYGAEALERAGILPDPSKPSKRSFIEDLLFNKVHEYGAEALERAGAKFVAAWTLKAAAGGGS
C4 adjuvant = Ribosomal protein adjuvant
EAAAKMAKLSTDELLDAFKEMTLLELSDFVKKFEETFEVTAAAPVAVAAAGAAPAGAAVEAAEEQSEFDVILEAAGDKKIGVIKVVREIVSGLGLKEAKDLVDGAPKPLLEKVAKEAADEAKAKLEAAGATVTVKEAAAKAKFVAAWTLKAAAGGGSQCVNLTTRTQLPPAYTNSFTRGVGGGSGDEVRQIAPGQTGKIADYNYKGGGSAKFVAAWTLKAAAGGGSILPDPSKPSKRSFIEDLLFNKVHEYGAEALERAGSYQTQTNSPRRARSVASQSHEYGAEALERAGAKFVAAWTLKAAAGGGS
C5 adjuvant = flagellin adjuvant
EAAAKMAQVINTNSLSLLTQNNLNKSQSSLSSAIERLSSGLRINSAKDDAAGQAIANRFTSNIKGLTQASRNANDGISIAQTTEGALNEINNNLQRVRELSVQATNGTNSDSDLKSIQDEIQQRLEEIDRVSNQTQFNGVKVLSQDNQMKIQVGANDGETITIDLQKIDVKSLGLDGFNVEAAAKAKFVAAWTLKAAAGGGSGDEVRQIAPGQTGKIADYNYKGGGSSYQTQTNSPRRARSVASQSGGGSAKFVAAWTLKAAAGGGSILPDPSKPSKRSFIEDLLFNKVHEYGAEALERAGQCVNLTTRTQLPPAYTNSFTRGVHEYGAEALERAGAKFVAAWTLKAAAGGGS

**Table 3:** Antigenicity, allergenicity, and solubility of the various vaccine constructs.

S. No	Antigenicity (Threshold > 0.4)	Solubility	Allergenicity (Threshold −0.4)
C1	0.4987	0.837445	−0.83923292
C2	0.5230	0.887539	−0.75524626
C3	0.5147	0.858435	−0.75971333
C4	0.4687	0.852533	0.13431533
C5	0.4846	0.520147	0.51140747

**Table 4:** Physiochemical properties of the vaccine constructs.

S. No	Number of Amino Acids	Molecular Weight (Daltons)	Theoretical pI	Aliphatic Index	GRAVY	Instability Index
C1	337	35,906.03	6.00	76.44	−0.431	39.46 (stable)
C2	223	23,438.58	9.88	64.13	−0.439	36.96 (stable)
C3	328	34,787.80	5.61	79.39	−0.399	44.66 (unstable)
C4	308	31,717.87	6.32	80.42	−0.140	28.73 (stable)
C5	353	37,181.20	9.01	78.39	−0.451	31.65 (stable)

**Table 5:** Docking results of TLR4, TLR7, and TLR8 with five vaccine constructs.

S. No	Solution Number	Global Energy (Kcal/mol)	Attractive VdW	Repulsive VdW	ACE	HB
TLR4/C1	268	−43.48	−41.07	20.13	0.39	−3.21
TLR4/C2	250	−48.43	−37.87	14.87	1.01	−3.09
TLR4/C3	753	−57.79	−34.68	24.66	−6.82	−6.15
TLR4/C4	58	−49.06	−32.34	3.16	6.95	−1.79
TLR4/C5	307	−37.22	−27.03	19.07	−2.02	−1.64
TLR7/C1	94	−65.88	−37.52	18.56	−7.02	−3.37
TLR7/C2	438	−47.16	−41.24	17.91	7.72	−3.18
TLR7/C3	256	−43.91	−26.80	6.60	2.39	−1.62
TLR7/C4	839	−55.98	−39.32	16.45	−3.15	−4.69
TLR7/C5	620	−47.34	−29.54	18.96	−3.22	−1.92
TLR8/C1	383	−60.24	−42.98	17.59	−0.09	−5.04
TLR8/C2	273	−57.44	−42.20	21.45	2.39	−6.75
TLR8/C3	973	−50.57	−29.17	24.59	−11.45	−1.43
TLR8/C4	280	−39.20	−34.23	10.77	5.27	−3.89
TLR8/C5	902	−60.34	−42.13	28.02	1.06	−2.06

**Table 6:** Protein–protein docking results of the vaccine construct and TLR7.

HADDOCK score	−132.1 +/− 7.3
Cluster size	45
RMSD from the overall lowest-energy structure	0.5 +/− 0.3
Van der Waals energy	−117.0 +/− 6.3
Electrostatic energy	−331.2 +/− 49.7
Desolvation energy	−75.8 +/− 6.4
Restraints violation energy	1269.5 +/− 72.06
Buried Surface Area	3552.9 +/− 125.7
Z-Score	−2.1
